# Incomplete duplication of a lower extremity (polymelia): a case report

**DOI:** 10.1186/1752-1947-8-184

**Published:** 2014-06-12

**Authors:** Nelson Montalvo, Ligia Redrobán, Victor Hugo Espín

**Affiliations:** 1 Pathology Service, Hospital Metropolitano, Av Mariana de Jesús s/n y Nicolás Arteta, Quito, Ecuador; 2 Genetics Service, Hospital Carlos Andrade Marín, Av 18 de Septiembre y Ayacucho, Quito, Ecuador

## Abstract

**Introduction:**

Polymelia, or congenital duplication of a limb, is an extremely rare entity in humans, with few cases reported in the literature.

**Case presentation:**

We present the case of a six-month-old Hispanic boy born with a lower limb bud on the left posterior thigh.

**Conclusion:**

The infant had a favorable outcome and evolution after surgical treatment of his supernumerary limb, with no after-effects or impairment whatsoever.

## Introduction

Polymelia (supernumerary limbs) is an extremely uncommon congenital entity rarely reported in humans [1,2], though it is frequently reported in animals [3-5]. Its pathogenesis is heterogeneous and includes incomplete separation of monozygotic twins [6-8].

## Case presentation

**Figure 1:**
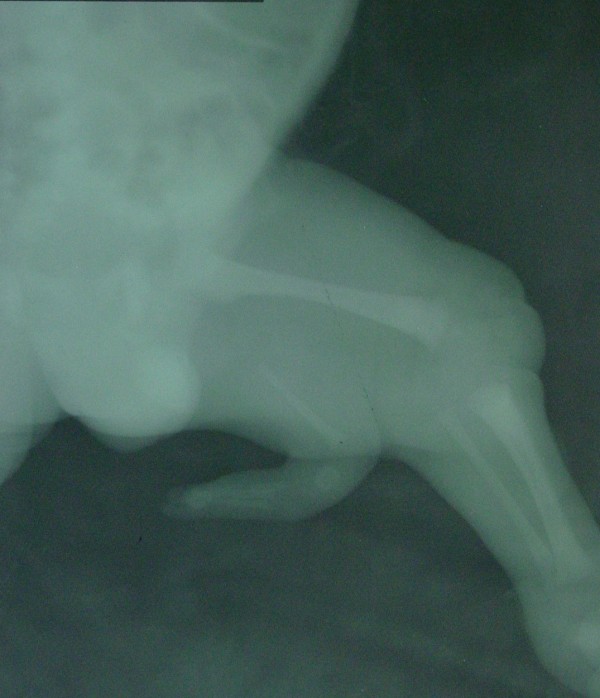
Plain film radiograph of left lower extremity shows an accessory lower limb bud on the posterior face.

**Figure 2:**
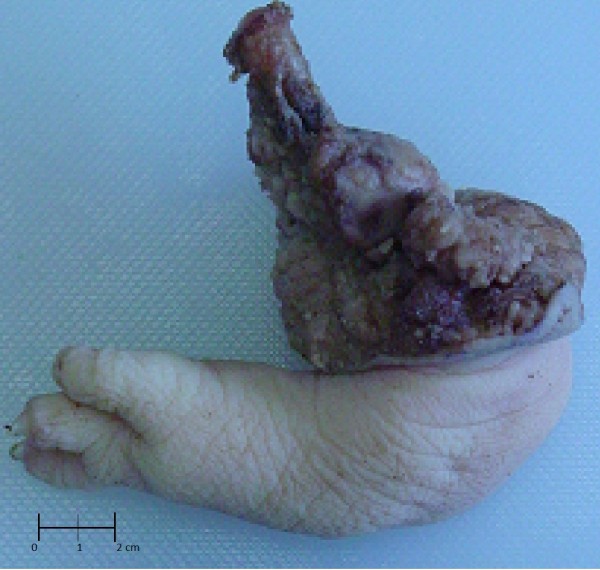
**Photograph of supernumerary limb with exposed bone segment.** Three digitiform formations are visible at the opposite end.

**Figure 3:**
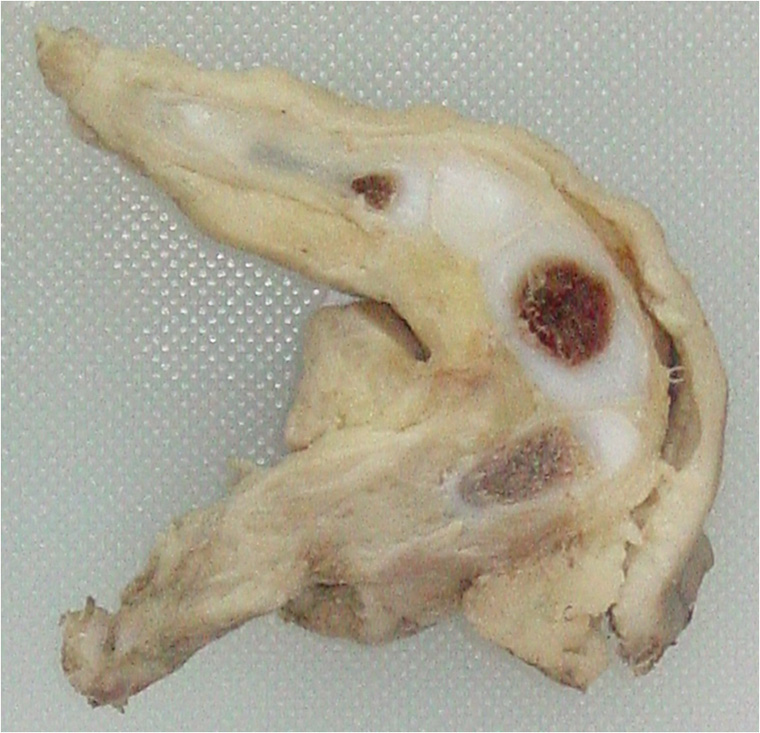
Photograph of the limb bud cut along its long axis showing islands of cartilage and centers of ossification surrounded by subcutaneous cellular tissue and skin.

**Figure 4:**
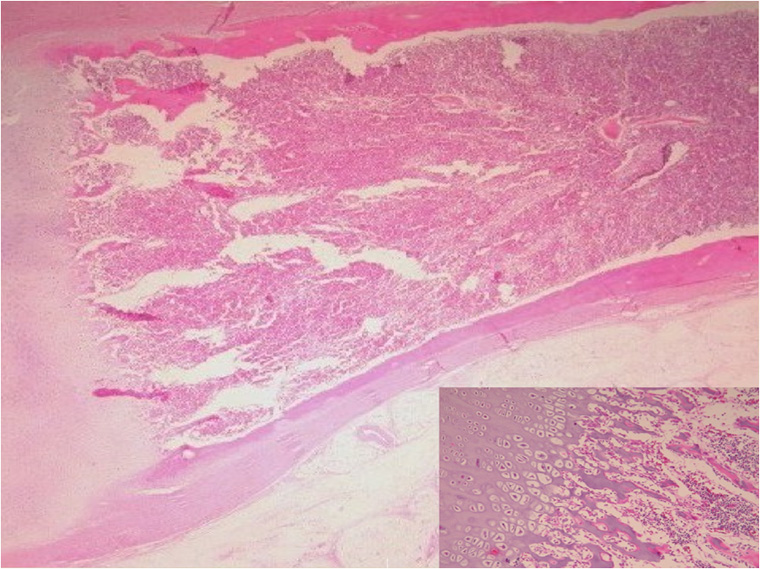
Histological specimen showing endochondral ossification in the diaphysis and the epiphysis (inset, 20x).

**Figure 5:**
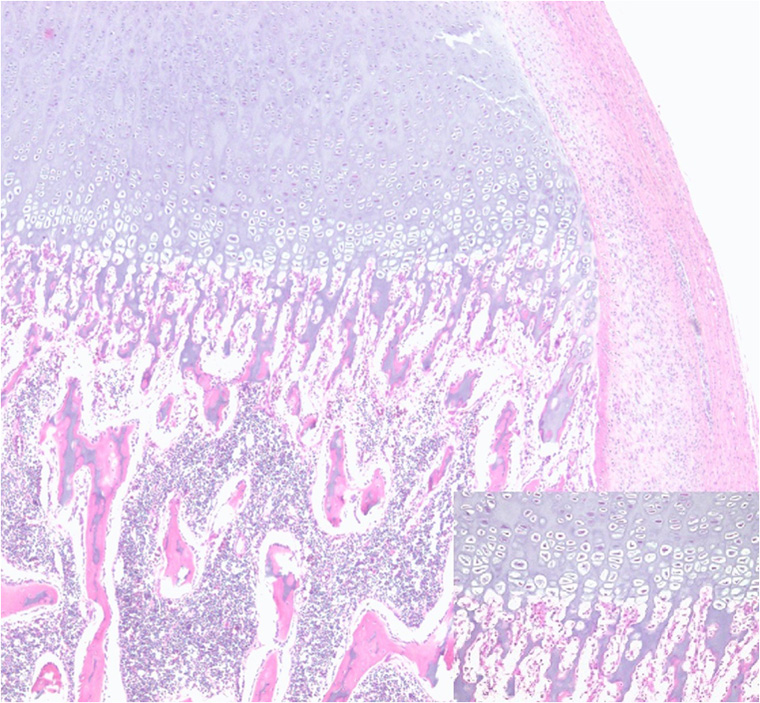
Histological analysis of supernumerary limb showing maturation of the cartilaginous growth plate in accordance with the infant`s age (inset, 40×).

Our case report concerns a six-month-old Hispanic first-born son of young, nonconsanguineous parents with no family history of hereditary diseases or major dysmorphology. The pregnancy passed without major complications and with no accidental or work-related exposure to genotoxic agents.The infant was born at term with normal anthropometry and no complications or major dysmorphic features except a limb bud, which was located on the posterointernal face of the left thigh (Figure 1).The undeveloped limb was surgically removed, and the 11-cm-long specimen was sent to the Pathology Service. The surgically removed end was bloody and had an exposed bone segment. There were three digitiform formations at the opposite end, two of which were joined together in a tweezer-like configuration. A kink with limited movement and covered with skin and abundant adipose tussue was observed in the central part (Figure 2). The surgical specimen was formalin fixed and paraffin embedded, cut at 4μm and subsequently stained with hematoxylin and eosin.The histopathological diagnosis was incomplete congenital duplication of the left lower extremity (polymelia). Histological analysis revealed diaphyseal endochondral ossification and cartilaginous epiphyseal plates maturing in accordance with the infant’s age (Figures 3, 4 and 5).

## Discussion

Polymelia (supernumerary limbs) is a rare congenital entity scarcely reported in humans, though not uncommonly in animals [1-4,9-12].

Limb development involves a very large number of genes [13]. One gene widely associated with the development of supernumerary limbs is the mouse mutant disorganization *Ds* gene [OMIM:223200] [9,14], which is a semidominant gene with variable penetrance in heterozygotes and lethality in homozygotes; 67% of heterozygotes have multiple defects and the rest have single defects, in which polymelia is prominent [10].

Limb development is a very complex process involving precise gene regulation fundamental to normal growth [11]. Findings in animal models have explained a great deal about these functions and have improved our understanding of the etiopathogeny of malformations, but more research is necessary to extend knowledge of these delicate processes. Surgical resection of the accessory limb at an early age is recommended in patients with supernumerary extremities [12,15].

## Conclusion

In our present case, the infant had a favorable outcome and evolution subsequent to surgical treatment of his supernumerary limb, with no sequelae or disability whatsoever to date.

## Consent

Written informed consent was obtained from the patient’s legal guardian(s) for publication of this case report and any accompanying images. A copy of the written consent is available for review by the Editor-in-Chief of this journal.

## Competing interests

The authors declare that they have no competing interests.

## Authors’ contributions

NM performed the histological examination and diagnosis of the patient. LR and VE conducted a thorough literature review of duplication of the limbs in the human tract and were the major contributors to the writing of the manuscript. All authors read and approved the final manuscript.
